# Supplementary material for: Trans-eQTLs Can Be Used to Identify Tissue-Specific Gene Regulatory Networks
Source: Curr Issues Mol Biol. 2025 Jul 29;47(8):594. doi: 10.3390/cimb47080594 (PMC12384777; doi:10.3390/cimb47080594)
Supplement: Supplementary file 1 [file cimb-47-00594-s001.zip › S1 Figure.pptx]

## Slide 1
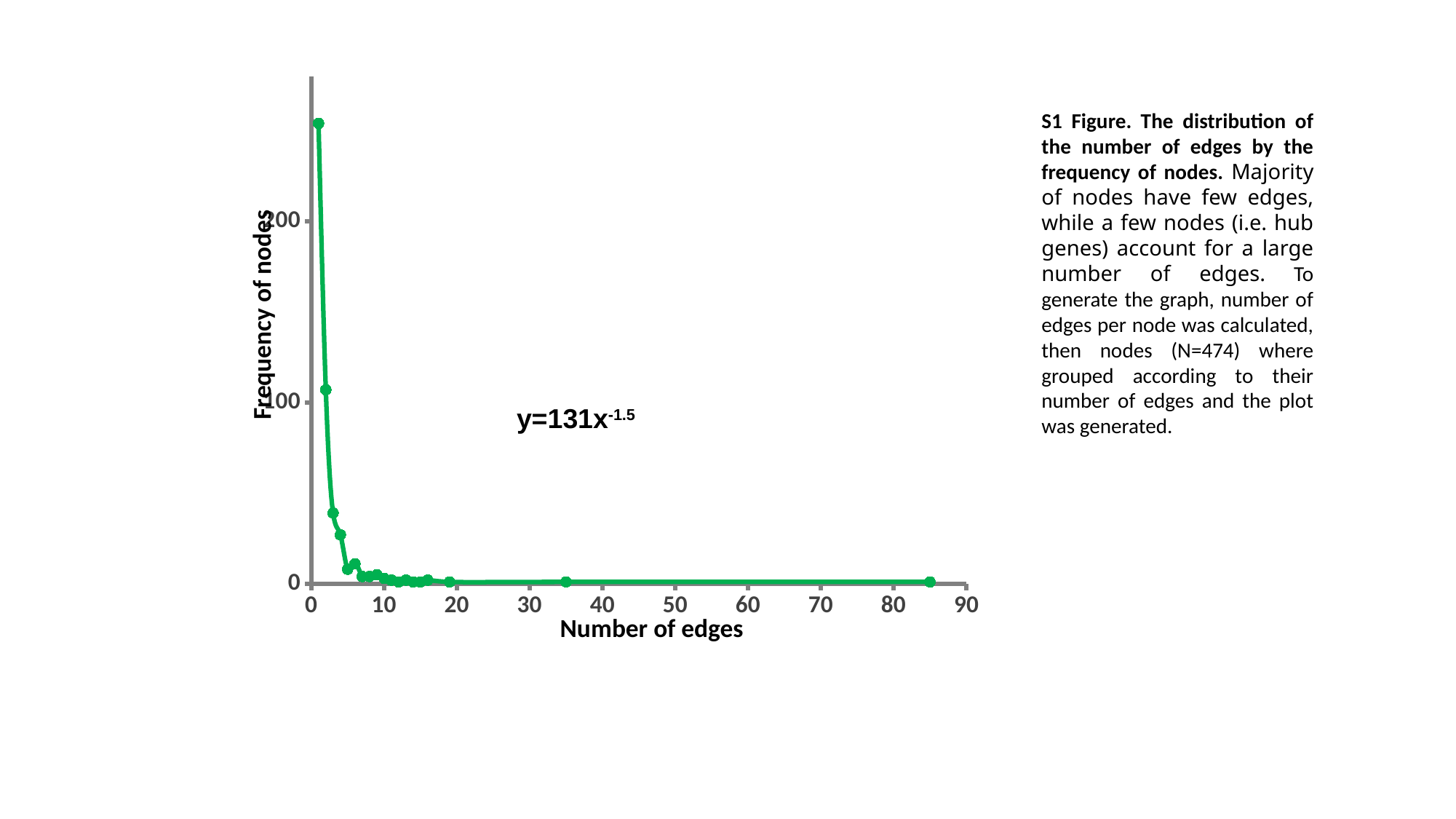

### Chart
| Category | |
|---|---|S1 Figure. The distribution of the number of edges by the frequency of nodes. Majority of nodes have few edges, while a few nodes (i.e. hub genes) account for a large number of edges. To generate the graph, number of edges per node was calculated, then nodes (N=474) where grouped according to their number of edges and the plot was generated.
Frequency of nodes
y=131x-1.5
Number of edges
