# Supplementary material for: Trans-eQTLs Can Be Used to Identify Tissue-Specific Gene Regulatory Networks
Source: Curr Issues Mol Biol. 2025 Jul 29;47(8):594. doi: 10.3390/cimb47080594 (PMC12384777; doi:10.3390/cimb47080594)
Supplement: Supplementary file 1 [file cimb-47-00594-s001.zip › S2 Figure.pptx]

## Slide 1
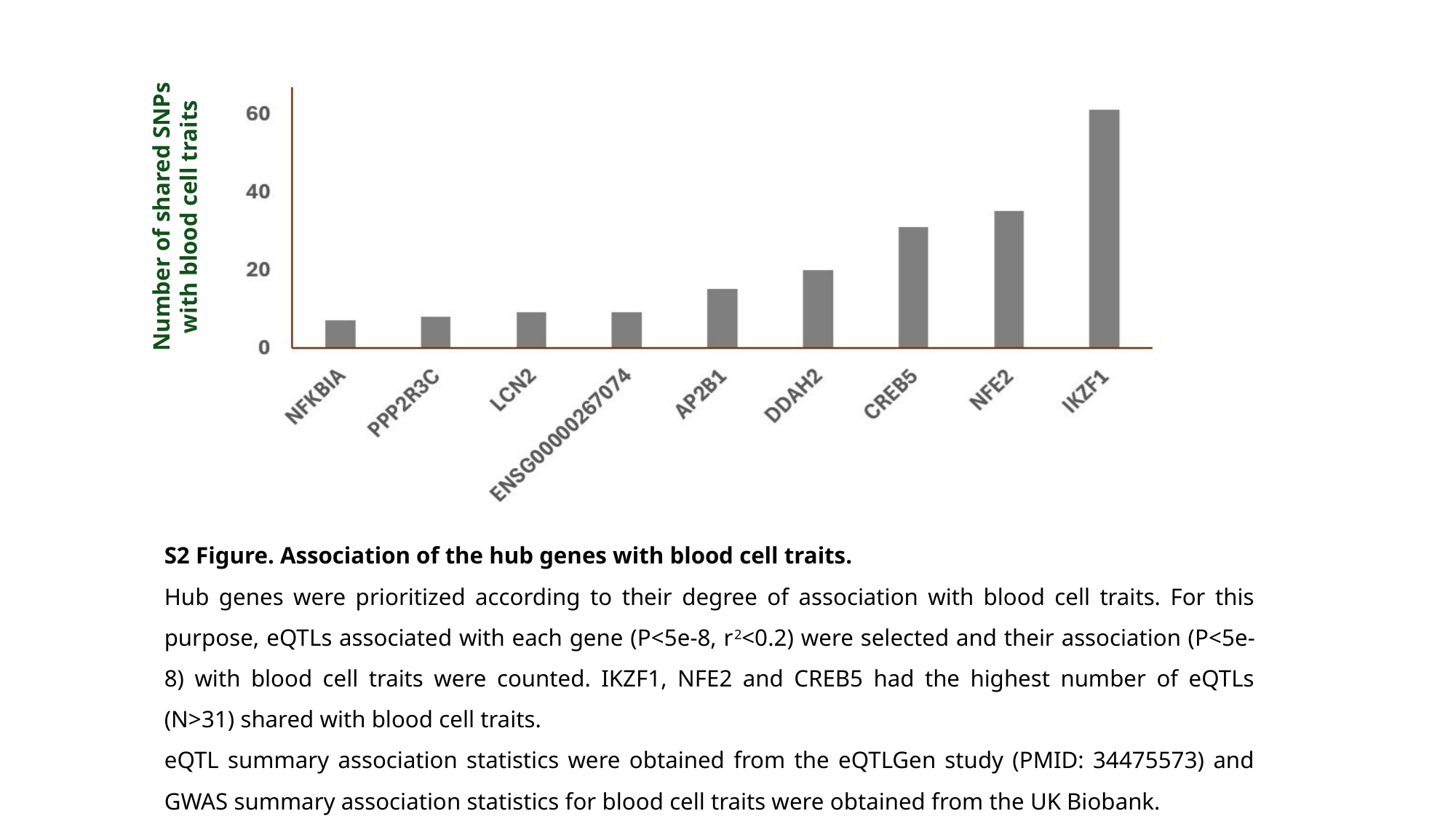

Number of shared SNPs with blood cell traits
S2 Figure. Association of the hub genes with blood cell traits.
Hub genes were prioritized according to their degree of association with blood cell traits. For this purpose, eQTLs associated with each gene (P<5e-8, r2<0.2) were selected and their association (P<5e-8) with blood cell traits were counted. IKZF1, NFE2 and CREB5 had the highest number of eQTLs (N>31) shared with blood cell traits.
eQTL summary association statistics were obtained from the eQTLGen study (PMID: 34475573) and GWAS summary association statistics for blood cell traits were obtained from the UK Biobank.
